# A direct shear apparatus for intact rock under dynamic moisture content

**DOI:** 10.1371/journal.pone.0272004

**Published:** 2022-08-23

**Authors:** Liyao Ma, Bin Hu, Kai Cui, Erjian Wei, Zhen Zhang, Zeqi Wang

**Affiliations:** 1 School of Resources and Environmental Engineering, Wuhan University of Science and Technology, Wuhan, Hubei, China; 2 Hubei Key Laboratory for Efficient Utilization and Agglomeration of Metallurgic Mineral Resources, Wuhan, Hubei, China; Sapienza University of Rome: Universita degli Studi di Roma La Sapienza, ITALY

## Abstract

Under field conditions, the moisture content of rock changes with the weather during prolonged creep. In order to investigate the effect of moisture content change on long-term shear strength and deformation behavior, a shear apparatus for intact rock was developed. Since three prefabricated holes are drilled in the upper part of the rock sample, the water injection device and the gas injection device can be used to inject water and gas into the rock sample alternately during the test to adjust the moisture content without removing the normal load and shear load. By using silicone gasket and seals in the shear box, fluid injection at a pressure of 5 MPa was achieved without leakage. Shear creep tests of argillaceous shale were conducted under both constant and dynamic moisture conditions, and the results were described by the Nishihara model. The experimental results revealed that there are significant differences in the long-term shear strength and deformation of argillaceous shale under different moisture content conditions. The proposed rock shear apparatus can advance the quantitative study of the shear creep properties of rock samples during moisture content changes and has certain practical application value for the prediction of engineering rock mass stability during rainfall.

## Introduction

In the field, the moisture content of rock changes with the weather conditions [1], and water would highly affect the physical (microstructures) and mechanical properties of rock [2–5]. Accurate evaluation of the shear properties of rock under dynamic moisture content conditions is essential for construction safety in geotechnical engineering. The shear-seepage test considering moisture content changes is an effective method of investigating the properties of rock, and the test results can provide important reference value for the prediction of landslide disasters.

Currently, many shear-seepage apparatuses have been developed to investigate the Shear seepage properties of rock joints [6–12]. The results obtained from these existing shear test apparatus provide important reference information for the development of an improved one. For instance, Yeo [13] improved the sealing performance by setting rubber hoops and rubber gaskets between the upper and lower shear boxes. Esaki [14] developed a shear-flow coupling test apparatus that can inject water from a prefabricated hole in the lower part of the rock sample during shearing. Further, Gutierrez [15] developed an unsealed shear apparatus to eliminate friction from the shear box. However, these studies aimed to reduce the fluid injection pressure in exchange for friction reduction, and modern rock shear-seepage coupled tests require the application of higher fluid pressures. Lee [16] developed a new test system using the material test system testing machine framework, which can apply a maximum fluid pressure of 0.49 MPa. Rong [17] used polyurethane materials to fabricate cushions and sealing rings to seal the shear box and achieved a good sealing effect. Jiang [18] introduced a newly developed apparatus to investigate the shear strength and flow behavior of rock joints, which can monitor the flow trajectory during testing. However, it remains difficult to study the effects of moisture content on intact rock during the shear test process by using the existing shear apparatus, and in many cases, continuous rock moisture content changes are critical to the stability of rock engineering structures.

Rainfall changes the moisture content of the weak interlayer of the slope, and the rise and fall of the reservoir water level change the moisture content of the bank slope [19], both of which lead to landslides. A compromise method of testing involves placing dry samples in a water atmosphere for some time to increase the moisture content before the shear test and then obtaining the relationship between the water soaking time and moisture content. Rock samples with constant moisture contents can be obtained by adjusting the soaking time according to this relationship [20, 21]; however, this method is not valid for studying the shear characteristics of rock samples under dynamic moisture content conditions. Therefore, it is necessary to develop a new shear apparatus to study the shear strength and deformation behavior of rock under changing moisture content conditions.

In this study, the rock sample structure and test equipment were optimized, and a device that can change the moisture content of the intact rock without removing normal and shear loads during testing was developed. Shear creep tests were conducted under both constant and dynamic moisture content conditions. Subsequently, the differences in the shear strength and deformation behaviors of the intact rock under both conditions were analyzed, and the Nishihara model was used to describe the creep characteristics of the rock. The proposed apparatus will help elucidate the shear characteristics of the intact rock under dynamic moisture content conditions.

## Development of the direct shear apparatus

### Design

For the real field condition, the moisture content of rock definitely changes with time during a rainfall. In addition, the rock strength would change with moisture content and the strengths of rock with different moisture content are the main areas of concern in the engineering practice. These rock instantaneous strengths could be tested in the condition of different constant moisture content. However, for a creep process of rock with long time, the moisture content of rock would change, and the test of the rock creeping property should be under the test condition with variational moisture content.

To realize changing moisture content during the rock shear creep test, we improved the test device and the structure of the rock sample and injected water or gas into the sample to change the moisture content. Injecting water can increase the moisture content of the rock sample, and injecting gas can reduce the moisture content. The most crucial aspects of improving the equipment are determining how to inject water or gas into the rock and how to seal the water or gas during the shear creep test properly.

**Fig 1:**
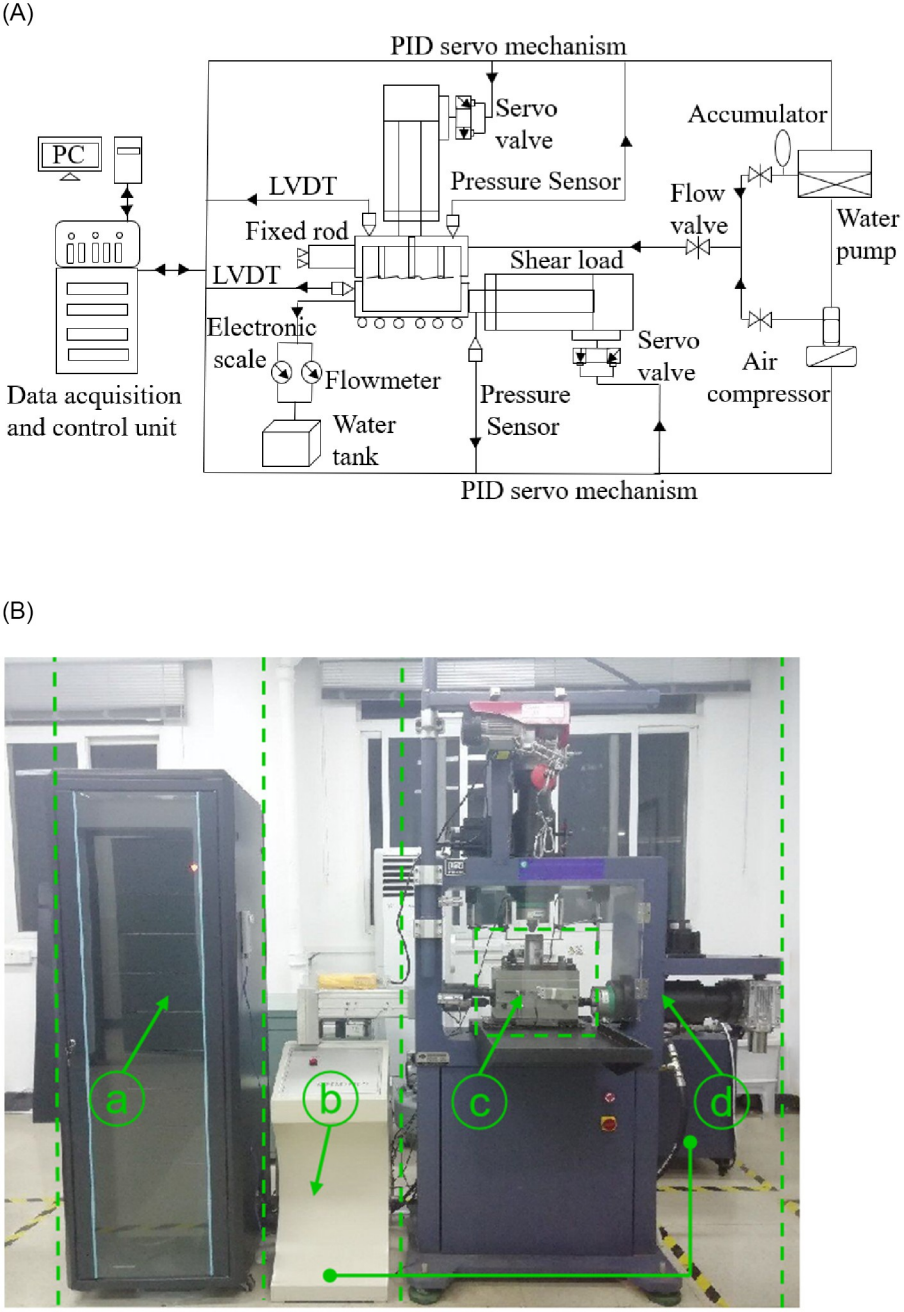
Proposed shear apparatus. (a) Simplified flowchart graph and (b) overall apparatus, which consists of the (a) data acquisition and control unit, (b) seepage loading unit, (c) servo stress loading unit, and (d) shear box.

Fig 1 depicts a simplified flowchart and the overall structure of the improved test apparatus, which includes three main units: a data acquisition and control unit (Fig 1a), seepage loading unit (Fig 1b), and servo stress loading unit (Fig 1c, as well as Fig 1d, the shear box).

### Fluid injection method

**Fig 2:**
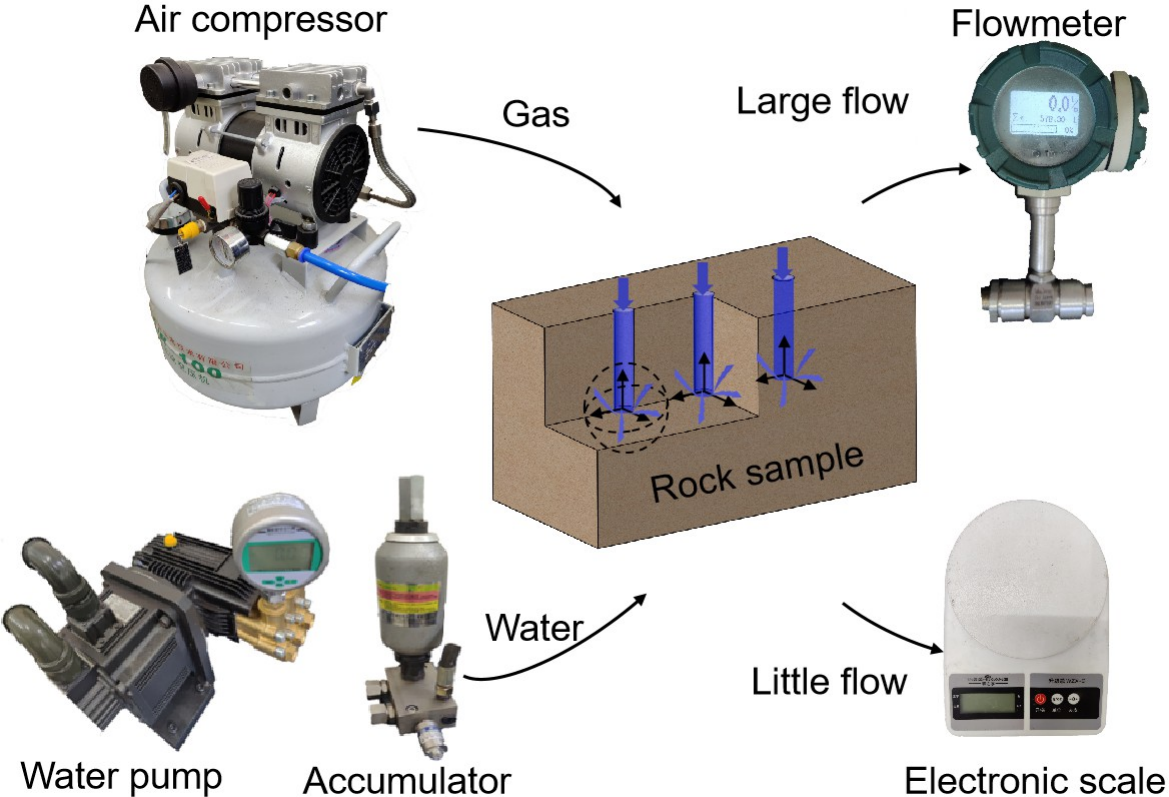
Seepage loading unit.

Intact rock contains numerous pores and micro-cracks, which can be used to store water or gas. A change in rock moisture content is essentially a change in the ratio of the water to gas content in pores and fissures, which can be adjusted by injecting water or gas into the rock. We systematically considered and designed the water and gas injection, transmission, and flow measurement devices and the structure of the rock sample to alter the moisture content of the rock efficiently during the shear creep test. The water and gas were pressurized and then introduced into the rock sample through a pipeline, as shown in Fig 2.

A water pump and an accumulator were used to pressurize and stabilize the water prior to injection. Both the outlet of the water pump and the outlet of the accumulator were provided with valves for adjusting the injected water pressure, which was 0–5 MPa, and the water pressure could be displayed in the digital pressure gauge in real time. An air compressor was used to pressurize and inject the air into the entire rock sample. The high-pressure gas injection drives the water out from the rock pores and fissures and reduces the moisture content of the rock sample. The injected gas pressure was 0–2 MPa. A high-pressure wire braided rubber hose that can withstand fluid pressure up to 34 MPa was used to transfer the water and gas. The rubber hose was equipped with a three-way valve to connect the shear box, water injection device, and gas injection device. When water was injected, the gas injection channel was closed, and vice versa. Two sets of flow measurement devices were designed to measure the amount of water seepage in the shear creep experiment. When the amount of seepage was small, an electronic scale was used to measure the water mass, and when it was large, a flowmeter was employed to measure the flow.

To speed up the rate of change of the moisture content in the rock sample, three prefabricated holes with diameters of 8 mm and depths of 37.5 mm were drilled at equal intervals along the center line on the upper surface of the 150 × 75 × 75 mm cuboid rock block. Pressurized water or gas was directly injected into the interior of the rock sample through the prefabricated holes. During the test, water and gas seeped out from the inside of the rock sample in the form of a radiant stream.

### Water or air sealing method

**Fig 3:**
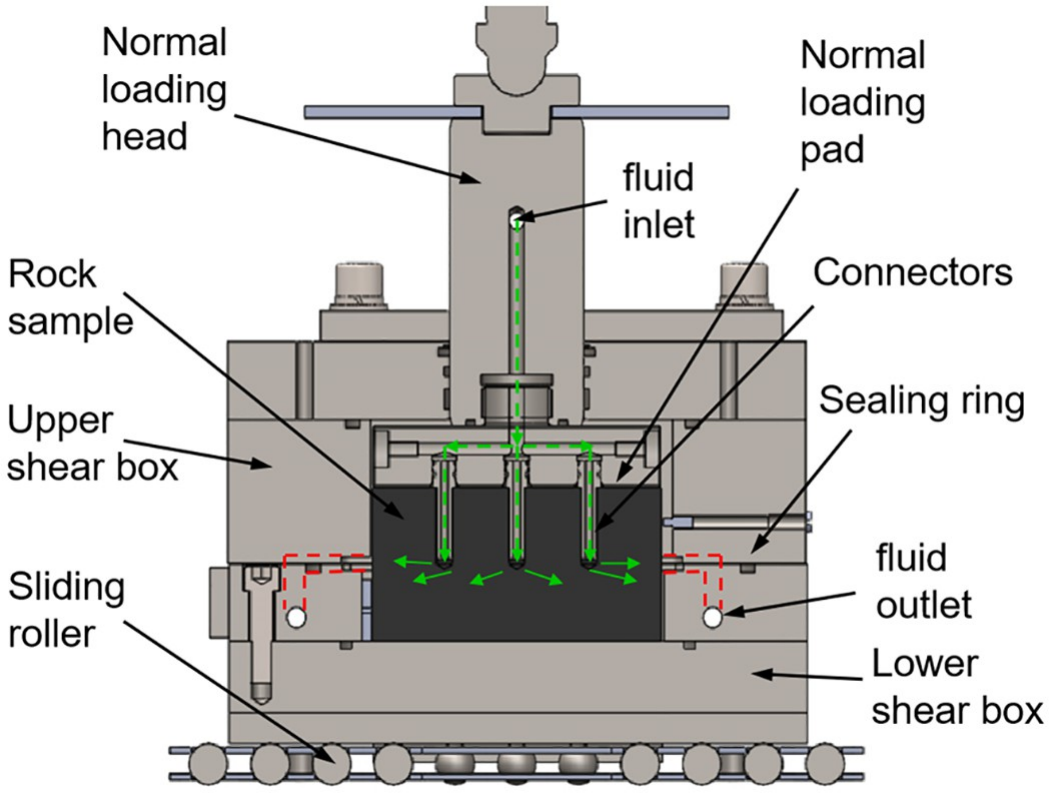
Structure of shear box.

The design and sealing material selection of the shear box is critical to changing the moisture content of the rock during the shear creep test. We designed a shear box comprising an upper shear box, a lower shear box, connectors, seal rings, a normal loading head, a normal loading pad, and a sliding roller, as shown in Fig 3. During the test, the lower shear box was pushed, and the upper shear box remained stationary due to contact with the fixed reaction rod. Water or gas was injected into the three prefabricated holes through the normal loading head, normal loading pad, and connectors, and two linear variable differential transformers were set on both sides of the shear box for displacement measurement. In addition, it was verified that polytetrafluoroethylene bronze composite material was suitable for manufacturing seal rings, and it was set in the shear box to prevent the leakage of injected high-pressure fluid [22].

**Fig 4:**
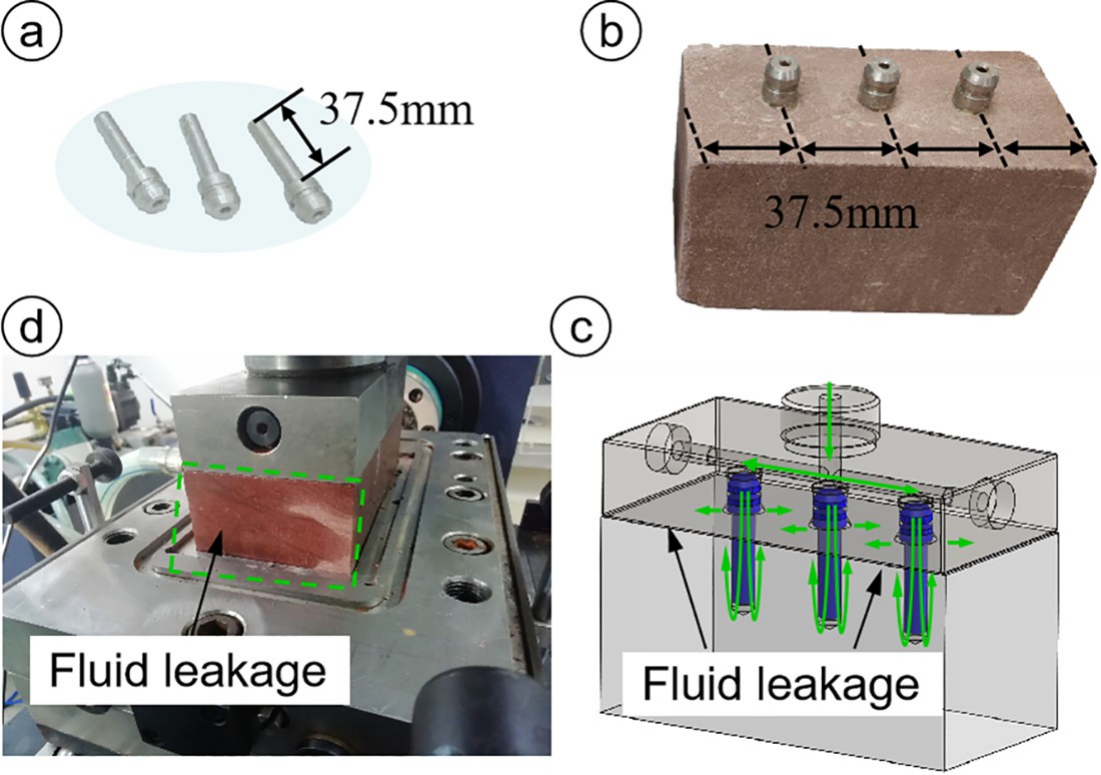
Sealing capacity with steel connectors. (a) Steel connector, (b) installation method of steel connectors, (c) flow path of injected water, and (d) sealing effect of steel connectors.

**Fig 5:**
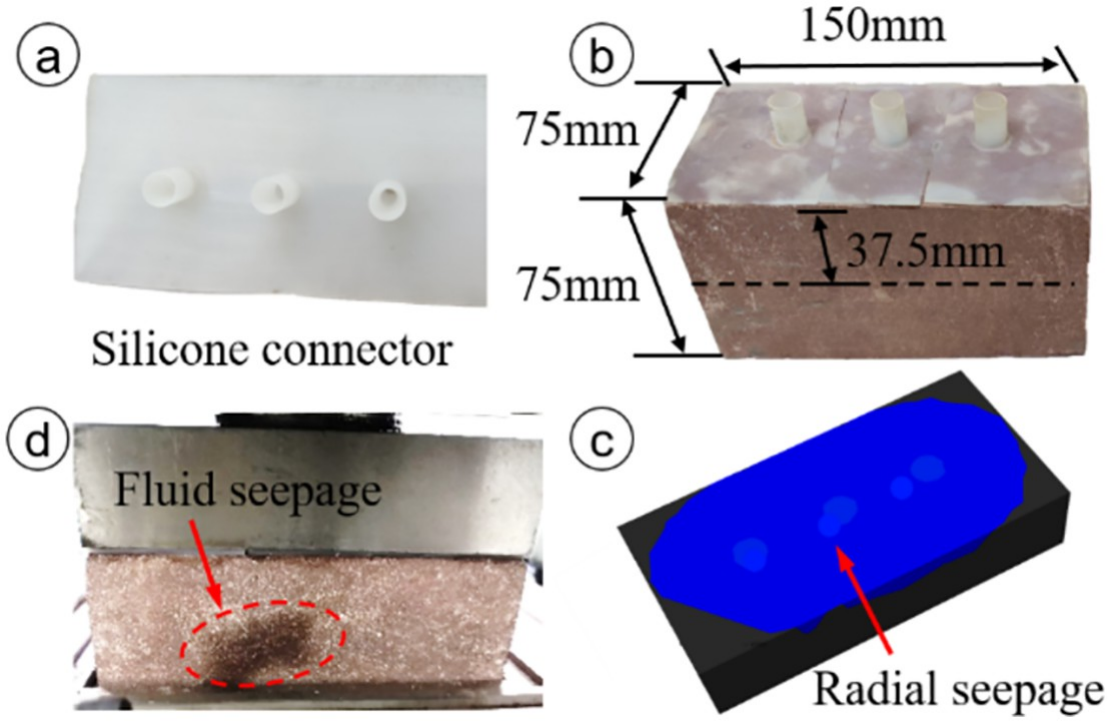
Sealing capacity with silica gel connectors. (a) Silica gel connectors, (b) installation method of silica gel connectors, (c) flow path of injected water, and (d) sealing effect of silica gel connectors.

Most important for the shear box is ensuring the sealing performance during testing. After comparison of the sealing effects of steel and silica gel connectors under 2 MPa of water pressure, flexible connectors comprising silica gel were selected to seal the high-pressure fluid. To facilitate observation of the fluid sealing effect, fluid was injected into red sandstone samples under constant normal load conditions. Prior to the test, the steel connectors and prefabricated holes of the rock sample were evenly coated with high-viscosity waterproof adhesive, and the steel connectors were tightly inserted into the holes. After injecting fluid, water leaked from the gap between the red sandstone and the normal loading pad in 2 min, as shown in Fig 4. The silica gel connectors were similarly glued to the red sandstone, and after water injection, no leakage was found in the gap, as shown in Fig 5. This finding proved that the sealing performance with the silica gel connectors was satisfactory.

### Important technical data

The presented rock shear apparatus can perform the following special tests: (1) rock shear-seepage and rock shear creep-seepage coupling tests under constant normal load and constant volume conditions. The testing method is as follows: first, apply a fixed normal load (CNL) or a fixed normal displacement (CV) to the predetermined value of the test scheme; then inject water (or gas) into the rock sample according to the test scheme to conduct a seepage test without removing the normal load; Finally, stop water (or gas) injection and apply shear load until the rock sample is sheared to failure, or the shear load is applied in stages until the rock sample is subjected to shear creep failure. (2) a shear creep-seepage coupling test during changing moisture content conditions. The testing method is as follows: first, apply a fixed normal load (CNL) or a fixed normal displacement (CV) to the predetermined value of the test scheme; then apply shear load in stages for the shear creep test; at the same time, water (or gas) can be injected into the rock sample to change moisture content until the rock sample undergoes shear creep failure.

Its main technical data are as follows: rock sample size,150mm*75mm*75mm;maximum normal load, 100 kN (100 N precision); maximum shear load, 100 kN (100 N precision); maximum normal displacement, 100 mm (0.01% precision); maximum normal displacement, 100 mm (0.01% precision); displacement loading rate, 0.001–10 mm/min; stress loading rate, 0.01–35 kN/s; maximum load retention time, 2000 h; water injecting pressure, 0–5 MPa; gas injecting pressure, 0–2 MPa; and fluid injection rate, 0–5 L/min (precision ±0.05%). In addition, the loading force, loading force speed, displacement, and displacement speed can be programmed, and the loading method can be switched at any time.

## Test verification

### Samples

**Fig 6:**
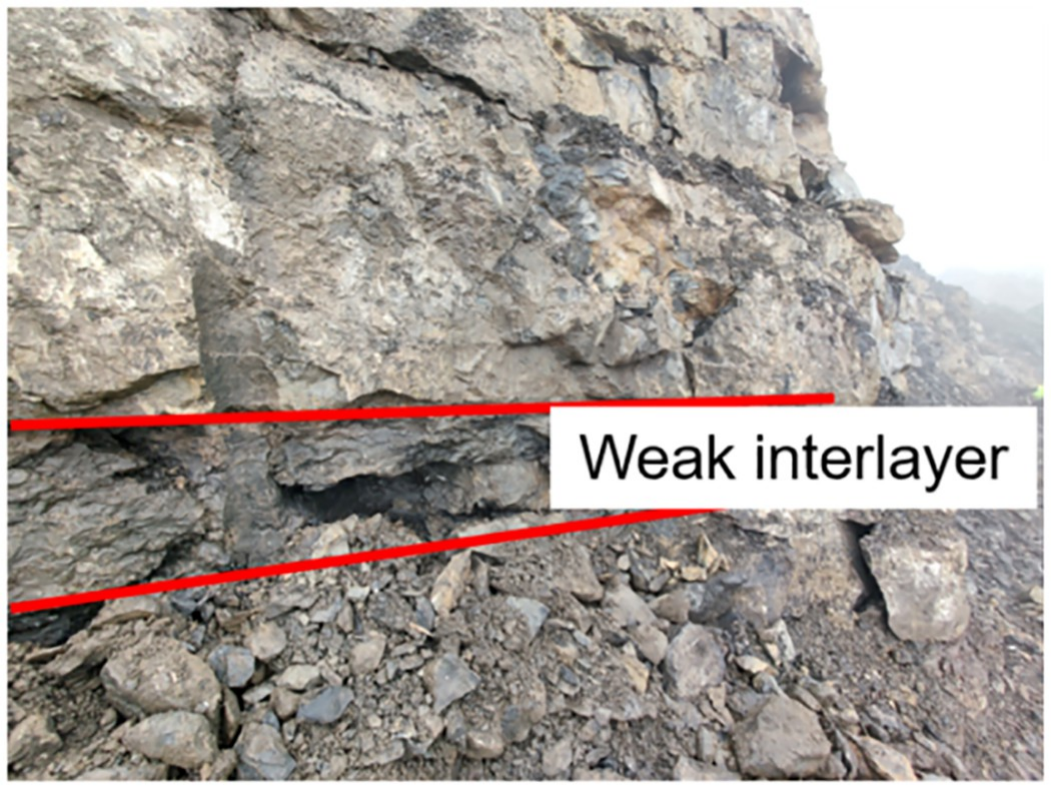
Graph of weak interlayers in mines.

**Fig 7:**
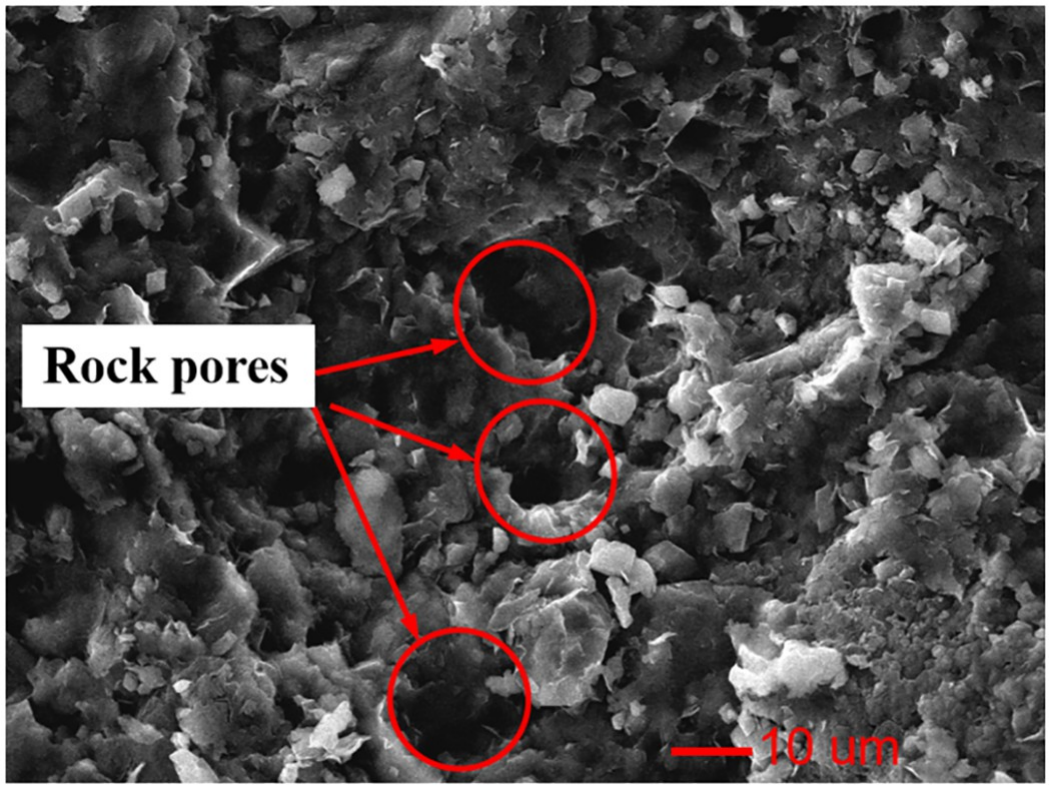
Electron microscope photograph of sample.

The argillaceous shale used to fabricate the test samples were obtained from the weak interlayer of the Esheng limestone mine in Emei, China, as shown in Fig 6. Through the identification of mineral components, it was found that the specimen consisted of CaO (38.03%), SiO_2_ (35.83%), MgO (13.03%), and Al_2_O_3_ (2.73%) and that the interior was porous, as shown in Fig 7. Many local landslide disasters in mines are caused by decreased strength and increased deformation of the weak interlayer under rainfall. The argillaceous shale specimen was processed into a 150 × 75 × 75 mm cuboid with a volume weight of 25.6 kN/m^3^. All six faces of the specimen were polished to a smoothness of 0.1 mm, dimension error of ±0.05 mm, and angle error of ±0.15°.

### Test scheme

Before the test, the argillaceous shale samples were placed in an oven at 105°C for 48 h. When the mass difference between measurements performed 24 h apart was less than 0.1%, the rock sample was regarded as dry, taken out, and cooled to room temperature 25°C. The relationship between moisture content and time was investigated by injection and immersion methods, respectively. To determine the relationship between the moisture content and injection time of the rock sample, a normal load of 0.6 MPa was applied to the dry rock sample and no shear load was applied. The injected water pressure was set to 2.0 MPa, and the continuous water injection time was 30 h. The moisture content was measured every 30 min until the weight of the sample remained unchanged. To determine the relationship between the moisture content of the rock sample and soaking time, a dry specimen was submerged in a container filled with purified water, then taken out and weighed every hour until the weight remained constant. In particular, water absorption is related to the number, size, degree of opening and closing, and distribution of rock pores. The saturated water absorption is obtained by the boiling water absorption method [23]. However, the water absorption should be obtained at normal temperature and pressure [24].

The moisture content *ω* of the sample was determined as follows:

$$\omega =\frac{m_{1}-m_{2}}{m_{2}}\times 100\%,$$ (1)

where *m*_1_ is the weight of the rock sample in the wet state and *m*_2_ is the weight of the rock sample in the dry state.

### Results

#### Water absorption of argillaceous shale samples

**Fig 8:**
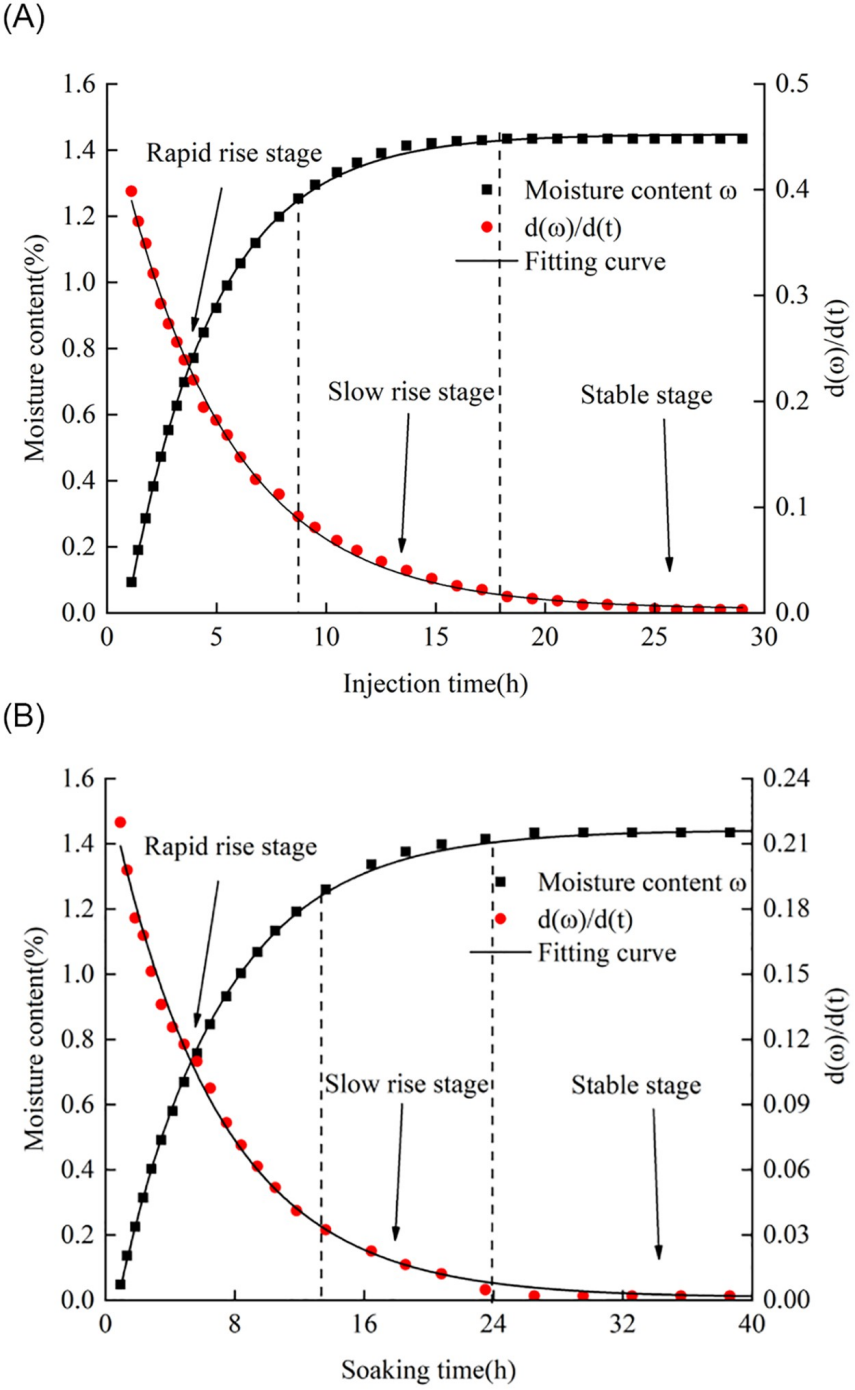
Variation of moisture content with (a) injection time and (b) soaking time.

Fig 8a shows the relationship between the moisture content of the argillaceous shale and the injection time, where the curves under different water injection pressures exhibit similar changes. According to the change rate of the moisture content of the rock sample, the curve could be divided into three stages: a rapid rise stage, a slow rise stage, and a stable stage. During the rapid rise stage, 0–8.5 h, water passed through and occupied the rock pores and fissures with better connectivity, and the moisture content increased with the injection duration. In the slow rise stage, 8.5–18 h, water gradually penetrated into small fractures and joints with narrow seepage channels and poor connectivity, and the moisture content increased at a slightly slower rate. In the stable stage, after 18 h, water occupied a large portion of the holes inside the rock, and the moisture content of the rock remained stable, which may indicate saturation. The moisture content at 0.5, 3.5, 7, and 18 h was 0.36%, 0.72%, 1.08%, and 1.44%, respectively.

Fig 8b shows the relationship between the moisture content of the argillaceous shale and the soaking time. This curve could also be divided into three stages based on the slope. During the first stage, 0–14 h, water was rapidly absorbed by the surfaces of the initially dried samples, and the moisture content increased with increasing soaking time. During the second stage, 14–24 h, a relatively complete permeation channel was formed inside the sample, and although the moisture content continued to increase, the growth rate was slightly slower than that during the first stage. During the third stage, 24–40 h, the water completely penetrated the sample, and the moisture content remained unchanged, which may mean that the sample reached a state of saturation. The moisture content at 1, 5, 10, and 26 h was 0.36%, 0.72%, 1.08%, and 1.44%, respectively.

We could fit the relationships between the sample moisture content and injection time and immersion conditions using the following exponential functions:

$$\text{y}=1.44–1.62\text{exp}\left(-\text{x}/6.40\right)\;{\text{R}}^{2}=0.99$$ (2)

$$\text{y}=1.44–1.81\text{exp}\left(-\text{x}/4.23\right)\;{\text{R}}^{2}=0.99.$$ (3)

#### Shear creep test results

We performed shear creep tests under constant and dynamic moisture content conditions. The shear creep test of the argillaceous shale with differing moisture contents was conducted under constant temperature and humidity conditions, and the shear stress of the rock was 0.8 MPa under normal stress of 0.6 MPa.

**Fig 9:**
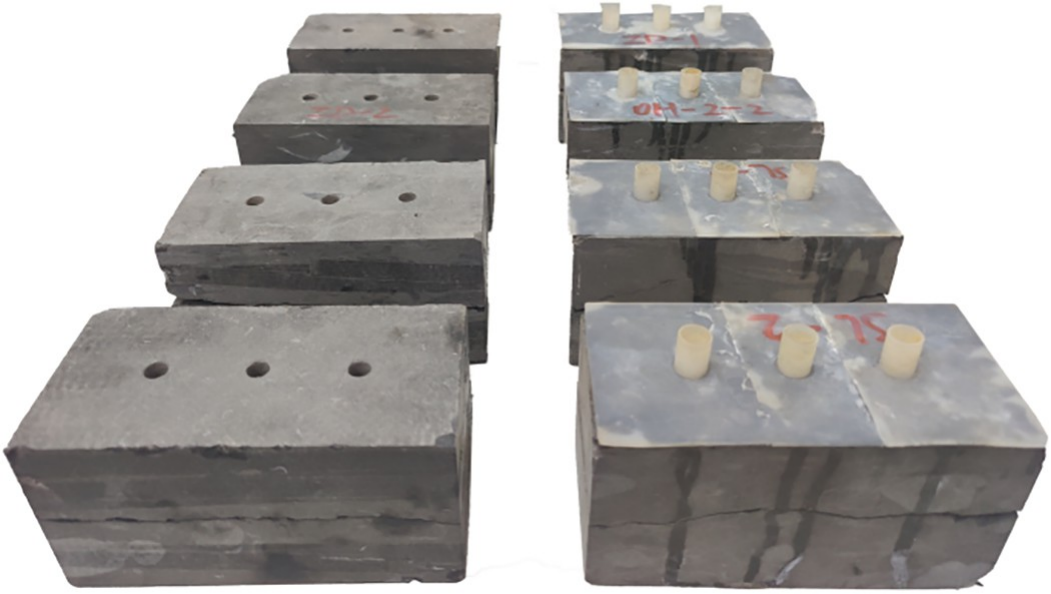
Graph of shear creep failure specimens.

**Fig 10:**
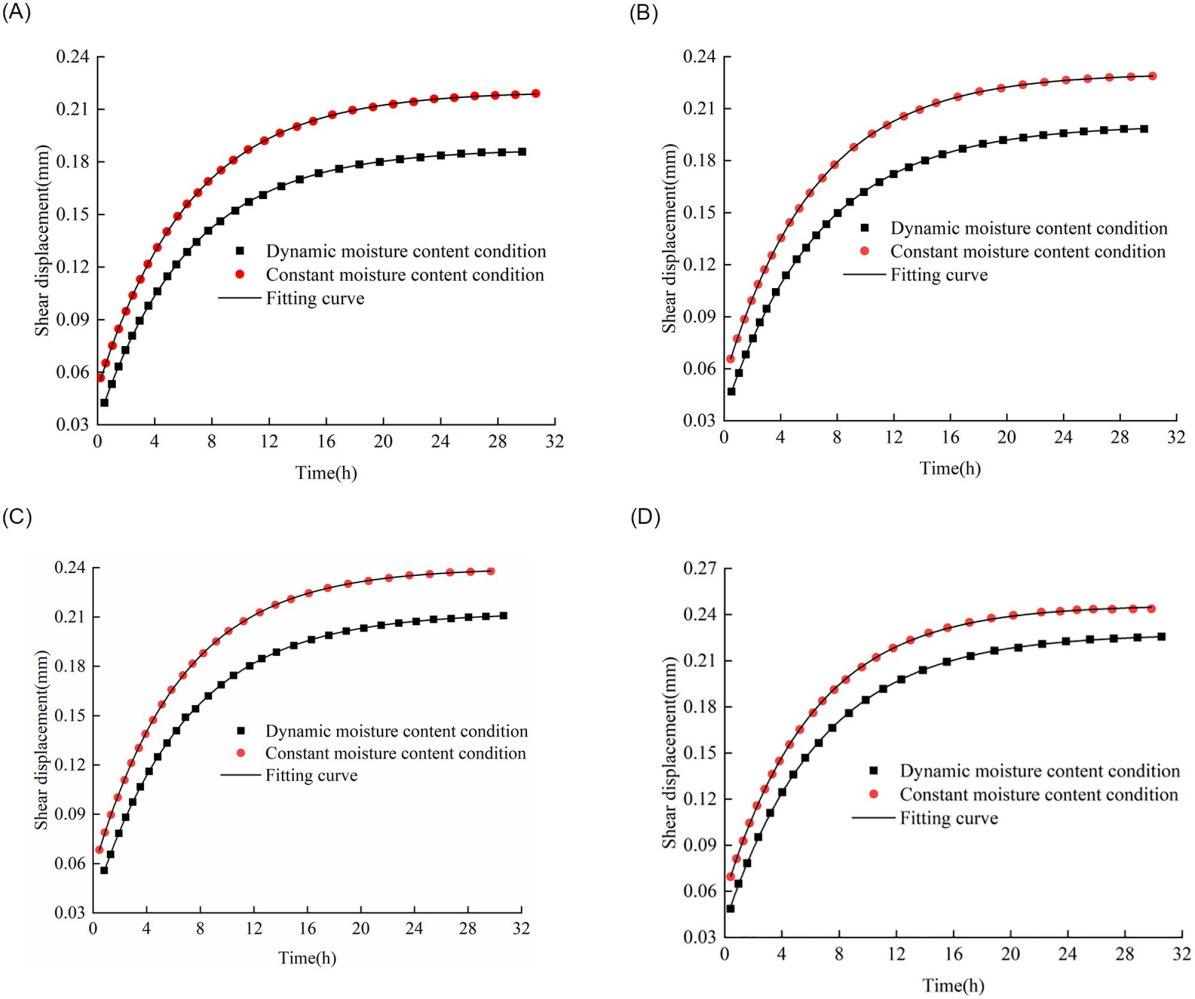
Shear creep test data and fitting curves under different moisture content conditions. Curves corresponding to peak moisture contents of (a) 0.36%, (b) 0.72%, (c) 1.08%, and (d) 1.44%.

Fig 9 depicts the samples after shear creep failure. One set of samples with silica gel joints was used for the shear creep test under injection conditions, and the other set was used under immersion conditions. Under the water injection conditions, each sample was in a dry state prior to shearing, and 2 MPa water was injected during the test. The moisture content increased continuously during the shearing process, after which the moisture content remained unchanged. Under the immersion conditions, the moisture content of each sample was increased to a predetermined value prior to shearing and remained constant during the test. We divided the experimental results in Fig 10 into two stages: a deceleration creep stage and a stable creep stage. In the deceleration creep stage, the shear deformation of the sample increased continuously, but the deformation rate gradually decreased. In the stable creep stage, the shear deformation of the sample remained almost unchanged, and the deformation rate approached zero.

The classical Nishihara model is widely used to describe the creep deformation characteristics of soft rock under low shear stress levels, as shown in Eq (4). It includes Hooke, Kell, and ideal viscoplastic bodies in series. In this study, the classic Nishihara model was used to describe the shear creep characteristics of the argillaceous shale under constant and dynamic moisture content conditions. The Levenberg–Marquardt optimization iterative algorithm was employed to identify the parameters of the classical Nishihara model [25], and Tables 1 and 2 summarize the identification results of the various parameters:

$$\varepsilon (t)=\frac{\tau }{G_{0}}+\frac{\tau }{G_{1}}\left[1-\text{exp}\left(-\frac{G_{1}}{\eta_{1}}t\right)\right]\left(\tau <\tau_{s}\right).$$ (4)

**Table 1:** Nishihara model parameters under dynamic moisture content conditions.

Peak dynamic moisture content (%)	Normal stress (MPa)	Shear stress (MPa)	*G*_0_	*G*_1_	*η*_1_
			(MPa/mm)	(MPa/mm)	(MPa·h/mm)
1.44	0.60	0.80	20.00	4.32	30.10
1.08			21.27	4.56	31.28
0.72			22.60	4.84	32.67
0.36			23.57	5.18	33.52

Peak dynamic moisture content listed in Table 1 can be regarded as the highest moisture content when the rock sample fails according to Fig 8.

**Table 2:** Nishihara model parameters under constant moisture content conditions.

Constant moisture content (%)	Normal stress (MPa)	Shear stress (MPa)	*G*_0_	*G*_1_	*η*_1_
			(MPa/mm)	(MPa/mm)	(MPa·h/mm)
1.44	0.60	0.80	13.07	4.28	27.19
1.08			13.58	4.41	28.73
0.72			14.36	4.57	29.61
0.36			15.26	4.76	30.98

Tables 1 and 2 summarize that, under either dynamic or constant moisture content conditions, the instantaneous shear modulus *G*_0_, viscoelastic shear modulus *G*_1_, and shear viscosity coefficient *η*_1_ decreased with increasing moisture content. Compared with the test data under dynamic moisture content conditions, when the moisture content was 1.44%, 1.08%, 0.72%, and 0.36%, the shear creep displacement increased by 8.01%, 12.86%, 15.39%, and 17.90%, respectively. Therefore, the moisture content change significantly affected the shear creep characteristics of the rock samples, which also proved that the newly developed shear instrument could well reflect the shear creep characteristics under changing moisture content conditions.

**Fig 11:**
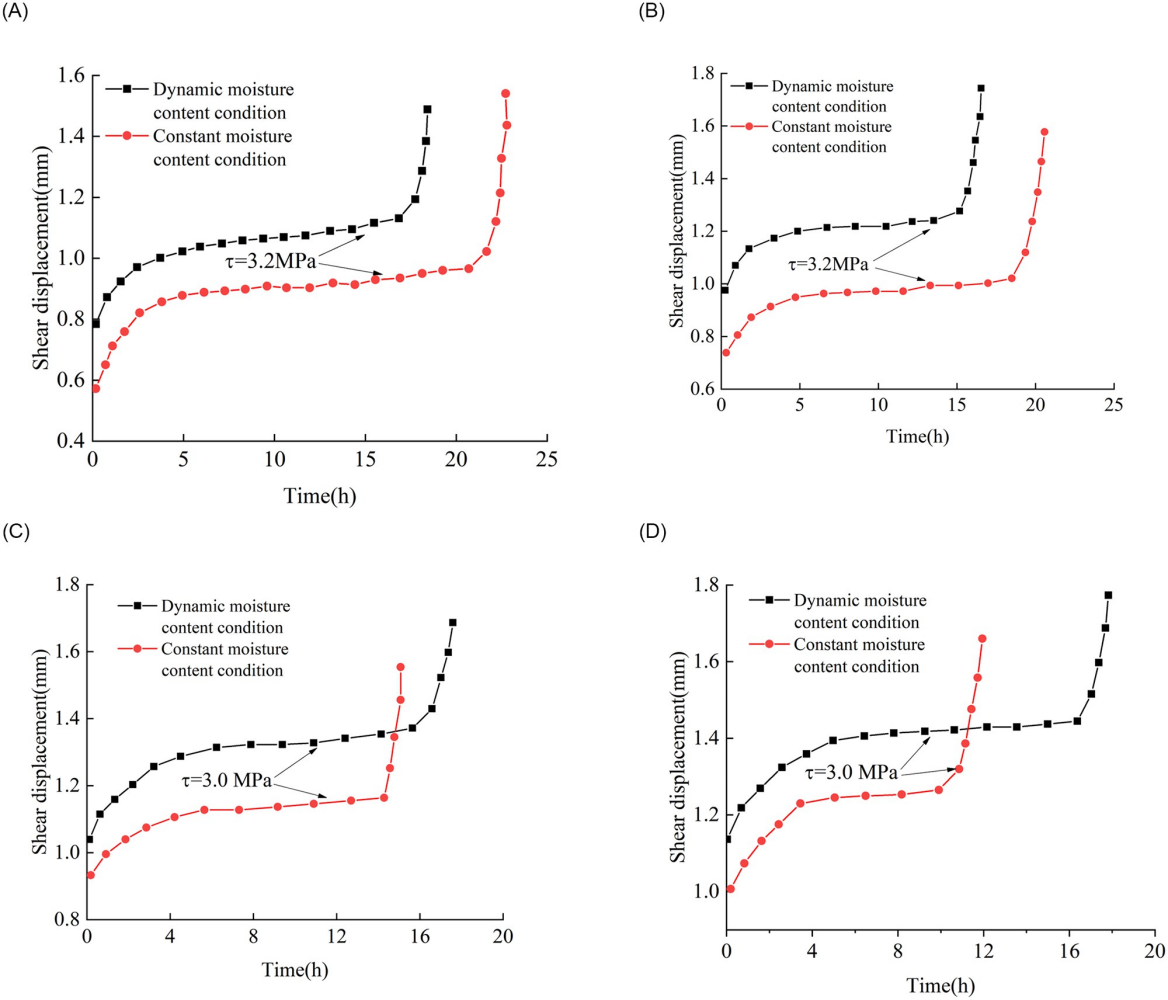
Shear creep failure test data under different moisture content conditions. Curves corresponding to peak moisture contents of (a) 0.36%, (b) 0.72%, (c) 1.08%, and (d) 1.44%.

It can be seen from Fig 11 that the rock samples undergo three stages when the shear creep failure occurs under the last shear stress: a deceleration creep stage, stable creep stage, and accelerated creep stage. The higher the moisture content of shale, the lower the strength and the greater the displacement at shear creep failure, which means that the increase in moisture content has a certain softening effect on argillaceous shale.

## Discussion

Until now, the characteristics of the changes in rock shear mechanical behaviors under varying moisture content conditions have not been clarified. The results of the shear creep experiments performed in this study demonstrate that even if the peak dynamic moisture content and fixed moisture content are the same, *G*_0_, *G*_1_, and *η*_1_ of the studied argillaceous shale still have significant differences. This phenomenon can be explained by several mechanisms through which water weakens rock, including pore water pressure, capillary tension, and water distribution. In the direct shear test under constant moisture content conditions, even if a slow shear rate was used, the increase in rock pore pressure was not obvious [26, 27]. Therefore, we believe that with a constant moisture content, the influence of increases in pore pressure on the strength of the rock can be ignored in the shear creep test; Zhou et al. reached the same conclusion [28]. However, under dynamic moisture content conditions, the degree of influence of the pore water pressure and capillary tension on the shear properties of rock require further investigation. In addition, in the shear creep test under varying moisture content conditions, the differences in the shear characteristics of the specimens may also be attributed to the differences in the water distribution [29] and the moisture content change process. First, after an argillaceous shale sample was soaked in water for a short duration; the inside of the sample remained dry because water migrated from its surface to its core. After injecting water into the prefabricated hole for some time, the moisture content of the internal part of the sample increased first, because the water migrated from the core to the surface; the corresponding data were neglected in prior studies that only considered the general concept of moisture content [30, 31]. Second, the moisture content of the argillaceous shale sample soaked in water remained stable during the test, and the weakening effect of water on the rock remained unchanged. In this regard, the moisture content of water-flooded argillaceous shale samples continued to increase, and the argillization process was exacerbated by moisture content variations [32]. Overall, dynamic moisture content changes significantly affect the shear characteristics of the argillaceous shale, and the development of new test equipment will help quantify these effects. At the same time, the test using the new apparatus can more realistically simulate the shear seepage characteristics of the rock without removing the normal load in the field. The test results help to predict the deformation behavior, mechanical properties of the weak interlayer in the slope and the influence of water on the slope stability.

## Conclusions

In this paper, we proposed a rock shear test system to reflect the shear creep characteristics of rock under dynamic moisture content conditions. By injecting water or gas into three prefabricated holes, the moisture content of rock samples could be changed without removing the normal and shear loads. By adopting a specially designed shear box and flexible specimen connectors comprising silica gel, the water sealing capacity in the shear creep process was evidently improved. Even if the moisture content peak was the same, the weakening effect of the sample under constant moisture content conditions was more evident than that under dynamic moisture content conditions, as evidenced by *G*_0_, *G*_1_, and *η*_1_. In the shear creep test, the change in moisture content evidently had a nonnegligible effect on the experimental results, but the specific reasons for and mechanisms of this effect require further research. Nonetheless, the proposed shear test apparatus could provide important equipment support for the quantitative studies of rock shear strength and deformation behaviors under dynamic moisture content conditions. The experimental results are particularly important for analyzing and predicting the stability of engineering rock masses during rainfall.
